# Instrumental activities of daily living function and cognitive status among Chinese older adults: a serial multiple mediation model

**DOI:** 10.3389/fpubh.2024.1378979

**Published:** 2024-05-02

**Authors:** Sijie Huang, Wenjuan Zhong, Qingzhou Cheng, Yuxi Shuai, Jiahui Zhu, Jiawei Diao

**Affiliations:** School of Medicine and Health, Wuhan Polytechnic University, Wuhan, China

**Keywords:** IADL, sleep duration, social participation, depressive symptoms, cognitive status

## Abstract

**Objective:**

This study aimed to develop and validate a serial multiple mediation model to investigate the association between instrumental activities of daily living (IADL) function and cognitive status among older adults while exploring the underlying mechanisms.

**Methods:**

This cross-sectional study involved 3,665 individuals aged 60 years and older who participated in the China Health and Retirement Longitudinal Survey (CHARLS). A serial multiple mediation model was utilized to explore the direct and indirect relationship between IADL function and cognitive status and whether sleep duration, social engagement, and depressive symptoms mediated this relationship.

**Results:**

Decreased IADL function was associated with worse cognitive status [effect = −0.620, 95% CI: (−0.692, −0.540)]. Sleep duration, social participation (SP), and depressive symptoms all acted as mediators in the relationship between IADL function and cognitive status.

**Conclusion:**

This study found both direct and indirect associations between IADL function and cognitive status, providing new insights into the effective prevention and intervention of cognitive decline among older adults.

## 1 Introduction

The issue of health problems among older adults has gained increasing attention due to the acceleration of global aging. Cognitive decline is a prevalent health issue in this population, which can result in loss of wellbeing, dementia, and functional limitations (1–3). This imposes a significant burden of long-term care on families and society. Previous studies have mainly focused on the impact of older adults cognitive status on functional ability (4, 5). However, there is a paucity of research on functional ability as a predictor of cognitive status, especially when it involves the mediating role of some specific variables. Functional ability is usually assessed by basic activities of daily living (BADL) and instrumental activities of daily living (IADL). IADL covers more advanced skills for independent living compared to BADL (6), and a decline in IADL function often occurs earlier in people with declining physical functioning (7). This may mean that IADL function is an earlier predictor of cognitive decline in older people. Therefore, it is meaningful to further study the relationship between IADL function and cognitive status. The present study aimed to investigate how IADL function affects cognitive status by examining the mediating associations between sleep duration, social engagement, and depressive symptoms.

### 1.1 Adverse psychological and health outcomes of declining IADL functioning

Previous studies have shown that a decline in IADL function may be associated with some adverse health outcomes, such as depression and cognitive impairment (8–10). However, the underlying mechanisms are not well understood. According to the stress process theory (11), stress can impact an individual’s psychological outcomes either directly or indirectly by depleting some of the individual’s resources. In line with the theory’s indirect coping mechanisms of stress, sleep and social activity participation may be affected when older adults face the stressor of declining IADLs. This may be related to psychological issues such as depression, which can result in impaired cognitive status. A cross-sectional study revealed that IADL restriction was significantly associated with depression in older adults (12).

### 1.2 The mediating effect of sleep duration

The limitations of IADL function have been identified as risk factors for sleep disorders in older adults (13), which in turn may be linked to psychological and cognitive problems. Sleep disorders may impair cognitive functions by hindering neurodevelopment, emotion regulation, processing speed, and impeding memory consolidation and learning processes (13). Empirical studies have indicated that sleep duration is a significant risk factor for depressive symptoms and cognitive impairment (14–16). Moreover, moderate sleep duration may help prevent cognitive decline in older adults (17).

### 1.3 The mediating effect of social participation

Previous research has shown that functional ability is a crucial factor that impacts social participation (SP) among older adults (18). SP, in turn, has a significant effect on the emotions and cognition of older individuals. Based on the aging activity theory (19) and the buffering model (20, 21), the social interaction and social support obtained by older people in SP will help them eliminate negative emotions and maintain good mental health. A cohort study conducted in China found that a lack of SP predicted a higher risk of depression 4 years later (22). The results of another experiment also showed that active social engagement can effectively improve depression in older adults (23). In addition, a qualitative study revealed that the SP of Chinese older adults has a positive impact on their cognitive function (24).

### 1.4 Depressive symptoms as an emotional predictor of cognitive status

Depression, one of the most common mental health problems in older individuals, has been confirmed by many studies to be related to cognition. Persistent or worsening depressive symptoms may accelerate cognitive decline (25, 26). Based on these findings, we hypothesize that depressive symptoms may mediate the relationships between sleep duration/SP and cognitive status among older adults.

### 1.5 The present study

This study aimed to investigate the underlying mediating mechanisms between IADL function and cognitive status among Chinese older adults. Drawing on existing theoretical frameworks and empirical findings, we propose a serial multiple mediation model. Specifically, we hypothesized that IADL functioning in older adults may be directly or indirectly associated with cognitive status, while hypothesizing that indirect pathways may be mediated through sleep duration, social engagement, and depressive symptoms. Additionally, this model suggested that both sleep duration and social engagement have an impact on depressive symptoms, which in turn affect cognitive status.

## 2 Materials and methods

### 2.1 Sample

This is a cross-sectional study, and the data analyzed in this study came from the second-wave national survey (2013) of the China Health and Retirement Longitudinal Survey (CHARLS). The CHARLS is an ongoing nationwide longitudinal survey, first conducted in 2011, with participants followed up every 2 to 3 years (27). The survey involves residents aged 45 and older and their spouses in 150 counties and 450 villages in China; their information was obtained through face-to-face interviews. The second-wave national baseline survey was conducted from July 2013 to August 2013, involving 18,245 respondents. According to the United Nations definition of the older population in developing countries, we used 60 years as the cutoff age for inclusion in the sample (28). Participants without complete core information or other covariates were excluded from this study, and 3,665 participants aged 60 years and older were ultimately included in this study for analysis. In addition, the data collection of this study was approved by the institutional review board of Peking University.

### 2.2 Measures

#### 2.2.1 IADL

In CHARLS, IADL was measured by asking respondents if they had difficulty doing the following activities: doing household chores, cooking, shopping, managing finances, and taking medications. The answers were scored as follows: No, I do not have any difficulty = 1, I have difficulty but can still do it = 2, yes, I have difficulty and need help = 3, and I cannot do it = 4. The higher the total score corresponding to the answer, the worse the respondent’s IADL functioning was (29). Cronbach’s α was 0.85, and the value of KMO was 0.80, indicating that the scale used in this study was reliable.

#### 2.2.2 Sleep duration

Sleep duration was derived by self-report in a face-to-face interview. Respondents were asked to respond to the following two questions: (1) In the past month, how many hours of sleep did you get per night on average? (2) In the past month, how long was your average nap after lunch? Finally, the answers to these two questions were added together to get the sleep duration (30).

#### 2.2.3 Social participation

In the CHARLS, SP was measured by asking respondents whether they had participated in the following activities in the past month: interacting with friends; playing mahjong, chess, cards, or participating in community club activities; providing help to family, friends, or neighbors who do not live with you and who did not pay you for the help; attending sport, social, or other kinds of clubs; taking part in a community-related organization; doing voluntary or charity work; caring for a sick or disabled adult who does not live with you and who did not pay you for the help; attending an educational or training course; stocking investment; using the internet; and others. Each activity’s participation was scored as 1 point, and non-participation as 0 points (31), with the total score ranging from 1 to 11.

#### 2.2.4 Depressive symptoms

A 10-item Center for Epidemiological Studies Depression (CESD-10) Scale was used to identify the depressive symptoms of the participants over the last week. The CESD-10 scale consists of ten items, eight of which are negatively oriented and two of which are positively oriented. The items are all scored on a 4-point Likert scale (1 = indicating rarely or none of the time, 2 = indicating some or a little of the time, 3 = indicating occasionally or a moderate amount of the time, and 4 = indicating most or all of the time) (32). Higher scores on negatively oriented items indicate more severe depressive symptoms, while the opposite is true for positively oriented items. Therefore, we inverted the scores for the two positively oriented items in order to standardize the meaning of the scores for all the items, and then summed the scores for the 10 items to obtain the total score. The total score for the 10 items ranges from 10 to 40, with a higher score indicating increased severity. The CESD-10 showed high internal consistency in this study, with a Cronbach’s α of 0.81. The validity analysis showed that the KMO value was 0.88, indicating great validity.

#### 2.2.5 Cognitive status

Cognitive status was comprehensively assessed using the Telephone Interview for Cognitive Status (TICS-10), word memory, and graph drawing on four dimensions: orientation, attention, episodic memory, and visuospatial ability (33). Orientation and attention were measured using the TICS-10 through 10 questions about dates (year, month, and day), days of the week, seasons, and consecutive 5 times 100 minus 7 questions. The greater the number of correct answers, the higher the TICS-10 score, ranging from 0 to 10. The episodic memory score is the average of the immediate and delayed word recall scores and ranges from 0 to 10. Immediate recall was assessed by the number of words that respondents could recall immediately after the interviewer read out 10 Chinese nouns. Delayed recall was assessed by asking participants to recall as many words as possible after 4 to 10 min. Visuospatial ability was assessed by showing the respondents a picture and then scoring them based on whether they could draw a similar figure. A score of 1 was given to respondents who could draw a similar figure, and a score of 0 was given to respondents who could not. The sum of these cognitive scores represents the cognitive status of the respondents, with higher scores indicating better cognitive status.

### 2.3 Statistical analysis

We used *t*-tests and one-way ANOVA to compare the cognitive scores of respondents with different socio-demographic characteristics. Pearson’s correlation analyses were conducted to check correlations across all variables (IADL, sleep duration, SP, depressive symptoms, and cognitive status). The PROCESS macro for SPSS 26.0 was utilized to demonstrate this multiple serial mediation model (PROCESS Model 80). We controlled for age, marital status, hukou, sex, and education level as covariates when examining the serial mediation model. The bias-corrected 95% confidence interval (CI) was calculated using 5,000 bootstrapping resamples, and a two-tailed *p*-value of <0.05 was considered statistically significant in this study.

To test the hypothesized serial multiple mediation model, we conducted path analysis using the online analysis and visualization software SPSSAU (version 24.0).^1^ The chi-square statistic (*χ*^2^), the comparative fit index (CFI), the Tucker–Lewis index (TLI), and the root mean square error of approximation (RMSEA) with its 90% CI were used to assess the model fit. CFI and TLI values greater than 0.95 and RMSEA values less than 0.06 indicate goodness of fit (34). An absence of zero in the 90% CI indicates a significant indirect effect.

## 3 Results

### 3.1 Preliminary analyses

Table 1 shows the demographic information of the participants, whose ages ranged from 60 to 102 years (M_age_ = 68.68, SD = 7.26) with 1,824 female (49.8%) and 1,841 male participants (50.2%). Most respondents in the study were married (79.2%), reported to be illiterate (55.5%), and were not non-agricultural hukou (74.5%). We found that these socio-demographic factors were not statistically significant by comparing the means using independent *t*-test and ANOVA. Table 2 presents the means, standard deviations, and correlations of the main variables. Among the participants, the IADL scores ranged from 5 to 20 (M_IADL_ = 5.52, SD = 1.66), SP scores ranged from 1 to 7 (M_sp_ = 1.48, SD = 0.82), cognitive function scores ranged from 1 to 21 (M_cog_ = 11.8, SD = 4.11), sleep duration ranged from 0.5 to 15 h (M_sd_ = 7.04, SD = 2.02), and depression scores ranged from 10 to 40 (M_dep_ = 17.58, SD = 6.06).

**Table 1:** The characteristics of 3,665 participants and the results for comparing cognitive scores across categories of each variable.

Variables	*N* (%)	Mean	SD	F/t	p
Age				1.293	0.275
60–69	2,254 (61.5%)	11.72	4.08		
70–79	1,072 (29.2%)	11.73	4.20		
≥80	339 (9.3%)	12.10	3.99		
Marital status				1.318	0.188
Other	762 (20.8%)	11.93	4.32		
Married	2,903 (79.2%)	11.71	4.05		
Hukou				−1.086	0.278
Other	2,730 (74.5%)	11.72	4.13		
Non-agricultural	935 (25.5%)	11.88	4.06		
Sex				0.470	0.638
Female	1,824 (49.8%)	11.79	4.15		
Male	1,841 (50.2%)	11.73	4.07		
Education level				0.356	0.785
Illiterate	2,034 (55.5%)	11.799	4.102		
Elementary school	880 (24.0%)	11.780	4.093		
Middle school	466 (12.7%)	11.586	4.190		
≥High school	285 (7.8%)	11.705	4.090		

Comparisons of two categories were performed with unpaired *t*-test, and comparisons of ≥ 3 categories were performed with one-way ANOVA of multiple comparison.

**Table 2:** A correlation analysis of study variables.

Variables	IADL (score)	Sleep duration	Social participation	Depressive symptoms	Cognitive status
IADL (score)	1	−0.085^**^	−0.088^**^	0.265^**^	−0.250^**^
Sleep duration	−0.085^**^	1	0.035^*^	−0.313^**^	0.118^**^
Social participation	−0.088^**^	0.035^*^	1	−0.145^**^	0.241^**^
Depressive symptoms	0.265^**^	−0.313^**^	−0.145^**^	1	−0.304^**^
Cognitive status	−0.250^**^	0.118^**^	0.241^**^	−0.304^**^	1
M ± SD	5.515 ± 1.659	7.038 ± 2.017	1.484 ± 0.822	17.583 ± 6.065	11.759 ± 4.110

*n* = 3,665; ^**^ *p* < 0.01; ^*^ *p* < 0.05.

### 3.2 Correlation analyses of the study variables

We used the total score of IADL to analyze the relationship with other variables in this study. As shown in Table 2, IADL was negatively associated with sleep duration, SP, and cognitive status and positively associated with depressive symptoms. Sleep duration was positively correlated with SP and cognitive status and negatively correlated with depressive symptoms. Moreover, SP was positively associated with cognitive status, and they were both negatively associated with depressive symptoms.

### 3.3 Path analysis

**Figure 1:**
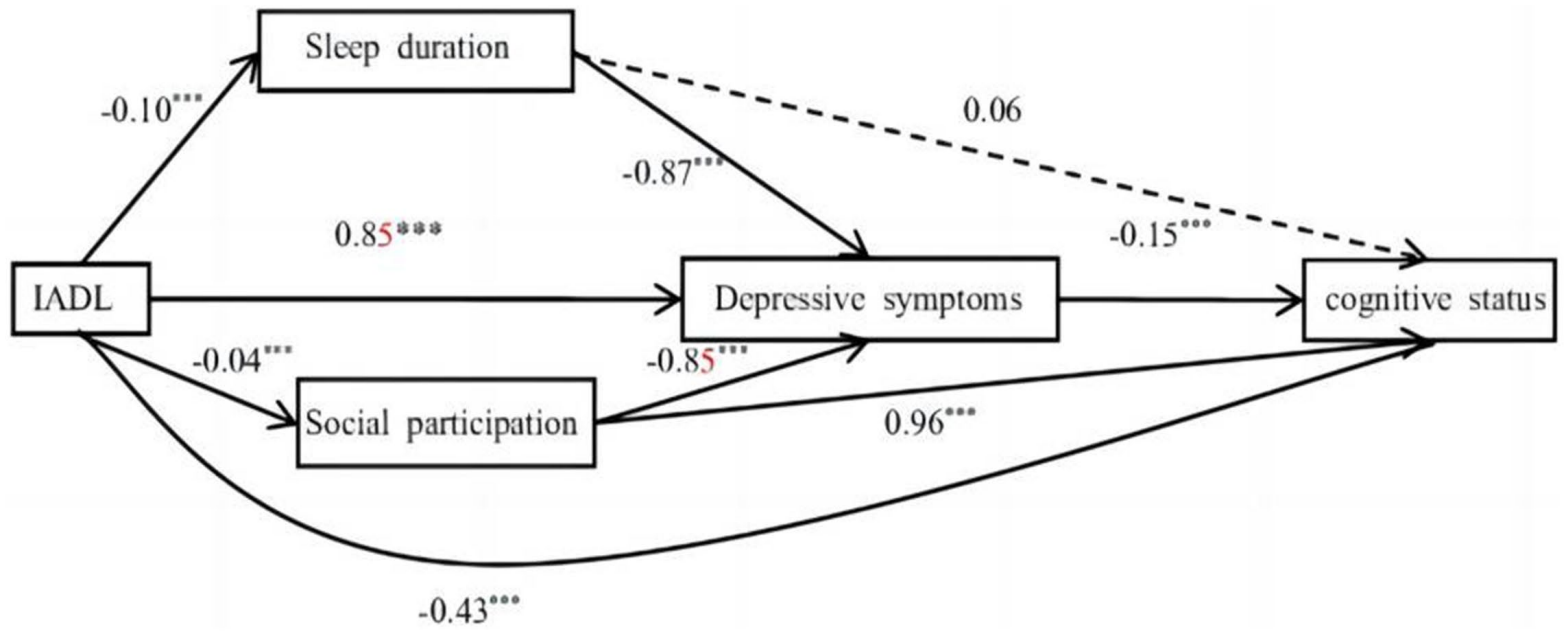
The serial mediation model. Solid lines indicate significant paths. Dashed lines indicate non-significant paths (^***^p < 0.001).

The hypothesized model showed good model fit, *χ*^2^ = 2.914, *p* = 0.088, CFI = 0.999, TLI = 0.986, RMSEA = 0.023, 95% CI [0.020, 0.055]. The final model and coefficients for each pathway are shown in Figure 1 and Table 3, respectively. The total effect [effect = −0.620, 95% CI: (−0.692, −0.540)], the direct effect [effect = −0.426, 95% CI: (−0.502, −0.350)], and the total indirect effect [effect = −0.193, 95% CI: (−0.230, −0.160)] of IADL on cognitive status were all significant. All indirect associations were statistically significant except for sleep duration, which did not directly mediate the relationship between IADL function and cognitive status. In addition, in Figure 1, the direct effect between IADL and cognitive status was more pronounced than any indirect effect. Among statistically significant indirect pathways from IADL function to cognitive status, the effect of Path 1 (IADL→depression→cognitive status) was the most pronounced [effect = −0.127, 95% CI: (−0.158, −0.100)]. This was followed, in descending order of impact, by Path 5 (IADL→sp.→cognitive status) [effect = −0.041, 95% CI: (−0.054, −0.028)], Path 3 (IADL→sleep duration→depression→cognitive status) [effect = −0.014, 95% CI: (−0.022, −0.006)], and Path 4 (IADL→sp.→depression→cognitive status) [effect = −0.006, 95% CI: (−0.008, −0.003)]. As shown in Figure 1, more severe IADL dependence was associated with poorer cognitive status (*β* = −0.43, *p* < 0.001), more severe depressive symptoms (*β* = 0.85*, p* < 0.001), shorter sleep duration (*β* = −0.10*, p* < 0.001), and less SP (*β* = −0.04, *p* < 0.001); both of the latter two were associated with more severe depressive symptoms (*β* = −0.87 and −0.85, both *p* < 0.001), which in turn were associated with poor cognitive status (*β* = −0.15, *p* < 0.001). Moreover, reduced SP resulting from decreased IADL function was associated with worse cognitive status (*β* = 0.96*, p* < 0.001).

**Table 3:** The results of serial mediation model analysis.

	Effect	Boot SE	Boot 95% CI
Total effect	−0.620	0.040	[−0.692, −0.540]
Direct effect	−0.426	0.039	[−0.502, −0.350]
Total indirect effect	−0.193	0.018	[−0.230, −0.160]
Path 1: IADL→depression→cognitive status	−0.127	0.015	[−0.158, −0.100]
Path 2: IADL→sleep duration→cognitive status	−0.006	0.004	[−0.015, 0.002]
Path 3: IADL→sleep duration→depression→cognitive status	−0.014	0.004	[−0.022, −0.006]
Path 4: IADL→sp.→depression→cognitive status	−0.006	0.001	[−0.008, −0.003]
Path 5: IADL→sp.→cognitive status	−0.041	0.007	[−0.054, −0.028]

The serial mediation model with PROCESS (Model 80). Paths with statistical significance at the *p* < 0.05 level are indicated in bold.

## 4 Discussion

The current study elucidated the relationship between IADL function and cognitive status among older Chinese adults. It formulated a serial multiple mediation model to examine the hypothetical underlying mechanisms. The findings validated the hypotheses that IADL function can be directly or indirectly associated with cognitive status, and that the relationship is mediated by sleep duration, social engagement, and depressive symptoms.

Our results confirmed that worse IADL function was associated with lower cognitive status. This is consistent with previous studies that found impairment in IADL function may occur at the stage of MCI and can be an early marker of cognitive decline (6, 35). The International Working Group on MCI has also recommended the inclusion of “preservation of basic ADLs, with some minimal impairment of complex instrumental functioning” in the diagnostic criteria (36). However, it has been suggested that some older adults with mild cognitive impairment have never experienced significant IADL decline (37).

The present study reached conclusions similar to those of a previous longitudinal study in Chinese adults, suggesting that IADL limitations are associated with failure to achieve optimal sleep duration (12). Influenced by Chinese Confucianism and Taoism, older Chinese people may view functional limitations as punishment. This perception may not only affect their image as wise elders and leaders in the family and society but also increase the burden of care for family members. Consequently, these long-term negative psychological implications may adversely impact their sleep (38, 39). Our findings align with the stress process theory that the decline of IADL function as a stressor may be associated with the depletion of self-resources, such as sleep disorders, resulting in negative emotions such as depressive symptoms (11). However, our study indicated that sleep duration did not directly mediate the relationship between IADL function and cognitive status in this particular model. Inconsistent with our study, most current studies have concluded that there is an inverted U-shaped relationship between sleep duration and cognitive function in older adults, where optimal sleep duration is associated with better cognitive performance (17, 40). The reasons for these different conclusions may lie in variations in the measurement of variables and the reporting of results. The sleep-cognition association may be linked to changes in the brain’s cortical structure and gray matter volume caused by inflammatory markers such as white blood cells (41, 42).

Our findings also offered support for previous theoretical and qualitative research indicating that functional decline in older adults impacts their SP (19), which in turn affects mental health and cognition (24, 25). The underlying physiological mechanism may be explained by the fact that reduced SP often predicts increased psychological stress. This increase in stress elevates catecholamines and glucocorticoids, leading to a series of physiological reactions such as the loss of dendritic spines and neuronal cell death, which can impair cognitive function (43).

Our study found that depressive symptoms significantly mediated the relationship between sleep duration/SP and cognitive status. These findings align with previous findings that inadequate sleep duration and a lack of social engagement are associated with depressive symptoms in older adults (15, 16, 23) and that depressive symptoms may be associated with cognitive decline (26). Some physiological and pathological mechanisms by which depressive symptoms may be related to cognitive decline have been proposed, such as sharing risk genes, vascular disease, hippocampal atrophy, increased deposition of amyloid-*β* plaques, inflammatory changes, inflammatory processes, and dysregulation of lipid transport (44, 45). From a psychological perspective, the relationship between depression and cognitive impairment can be explained by the fact that older adults focus on coping with the negative thoughts caused by depression while neglecting the cognitive reserve needed to deal with the problems (46).

Notably, we found that among statistically significant indirect pathways from IADL function to cognitive status, the effect of Path 1 (IADL→depression→cognitive status) was the most pronounced. This was followed, in descending order of impact, by Path 5 (IADL→sp.→cognitive status), Path 3 (IADL→sleep duration→depression→cognitive status), and Path 4 (IADL→sp.→depression→cognitive status). The stress process theory offers a potential explanation for these results; it suggests that the greater the use of personal resources and external support to cope with stress, the lower the negative impact on mental health outcomes (11). Additionally, Path 3 (IADL→sleep duration→depression→cognitive status) and Path 4 (IADL→SP → depression→cognitive status) share similar segments with Path 1 (IADL→depression→cognitive status), thereby reinforcing the effect observed in Path 1.

There were limitations in this study. First, measurements of the main variables were obtained through self-reporting, which may have resulted in biased results. Future research should attempt to introduce more objective and accurate measures to make the results more convincing. Second, our study was cross-sectional and therefore could not draw conclusions about causality or longitudinal trends. Future studies may need to be designed as longitudinal studies to determine the exact causal relationship between these variables. Third, the population sample investigated in this study was older adults in China. Due to regional differences, cultural differences, and other factors, it remains to be seen whether the conclusions of this study can be extended to older populations of other races, necessitating further research.

Despite the above limitations, the present study enriches the existing literature on the impact of IADL functioning on cognitive status in older adults and contributes to a deeper understanding of the underlying mechanisms. The results of this study also remind clinicians and caregivers to pay attention to changes in IADL function, a higher level of functional ability in older adults, so that cognitive decline and even dementia can be prevented in a timely and better way. In addition, healthcare providers could help older adults with functional decline adjust their mindsets and increase social interactions, with particular emphasis on maintaining optimal sleep duration in order to avoid adverse psychological conditions and cognitive decline.

In conclusion, this study provides evidence that IADL function is associated with cognitive status among Chinese older adults. The study also highlights the mediating role of sleep duration, social engagement, and depressive symptoms. Healthcare providers may consider targeting cognitive decline in older adults who exhibit declining IADL function. Strategies that could be used include improving sleep, encouraging participation in social activities, and addressing depressive symptoms, with particular emphasis on the latter. Future research on this topic could benefit from incorporating other variables to explore deeper and richer underlying mechanisms.

## Data availability statement

The original contributions presented in the study are included in the article/supplementary material, further inquiries can be directed to the corresponding author.

## Author contributions

SH: Writing – original draft, Writing – review & editing. WZ: Data curation, Methodology, Writing – review & editing. QC: Project administration, Supervision, Writing – review & editing. YS: Validation, Writing – review & editing. JZ: Conceptualization, Writing – review & editing. JD: Validation, Writing – review & editing.

## Footnotes

^1^https://spssau.com/indexs.html
